# Comb-Type Grafted Hydrogels of PNIPAM and PDMAEMA with Reversed Network-Graft Architectures from Controlled Radical Polymerizations

**DOI:** 10.3390/polym8020038

**Published:** 2016-02-01

**Authors:** Sheng-Qi Chen, Jia-Min Li, Ting-Ting Pan, Peng-Yun Li, Wei-Dong He

**Affiliations:** CAS Key Laboratory of Soft Matter Chemistry, Department of Polymer Science and Engineering, University of Science and Technology of China, Hefei, Anhui 230026, China; sqchen@mail.ustc.edu.cn (S.-Q.C.); lijiamin@mail.ustc.edu.cn (J.-M.L.); pting162@mail.ustc.edu.cn (T.-T.P.); pyli@mail.ustc.edu.cn (P.-Y.L.)

**Keywords:** hydrogels, stimuli-sensitive polymers, ATRP, RAFT, click chemistry, network-graft architecture

## Abstract

Dual thermo- and pH-responsive comb-type grafted hydrogels of poly(*N,N*-dimethylaminoethyl methacrylate) (PDMAEMA) and poly(*N*-isopropylacrylamide) (PNIPAM) with reversed network-graft architectures were synthesized by the combination of atom transfer radical polymerization (ATRP), reversible addition-fragmentation chain transfer (RAFT) polymerization and click chemistry. Two kinds of macro-cross-linkers with two azido groups at one chain-end and different chain length [PNIPAM–(N_3_)_2_ and PDMAEMA–(N_3_)_2_] were prepared with *N*,*N*-di(β-azidoethyl) 2-halocarboxylamide as the ATRP initiator. Through RAFT copolymerization of DMAEMA or NIPAM with propargyl acrylate (ProA) using dibenzyltrithiocarbonate as a chain transfer agent, two network precursors with different content of alkynyl side-groups [P(DMAEMA-*co*-ProA) and P(NIPAM-*co*-ProA)] were obtained. The subsequent azido-alkynyl click reaction of macro-cross-linkers and network precursors led to the formation of the network-graft hydrogels. These dual stimulus-sensitive hydrogels exhibited rapid response, high swelling ratio and reproducible swelling/de-swelling cycles under different temperatures and pH values. The influences of cross-linkage density and network-graft architecture on the properties of the hydrogels were investigated. The release of ceftriaxone sodium from these hydrogels showed both thermal- and pH-dependence, suggesting the feasibility of these hydrogels as thermo- and pH-dependent drug release devices.

## 1. Introduction

Stimuli-responsive hydrogels have the ability to change their volume, permeability and other properties in response to environmental stimuli such as temperature, pH, electric field and chemical substances [1,2,3,4,5,6]. Because of these useful performances, stimuli-responsive hydrogels have numerous applications, including drug delivery, biosensors, biocatalysts, tissue engineering and so forth [7,8,9,10,11,12,13]. Combining the thermal- and pH-responsive abilities into the hydrogels has opened more choices for modulating the hydrogel performance and broadening the practical applications [14,15,16,17].

For the practical applications of stimuli-responsive hydrogels, the high response rate is usually expected to be as fast as possible. To meet this target, several strategies have been proposed: (1) the utility of micro-hydrogels since their response rate is inversely proportional to the dimensional size of hydrogels [18,19]; (2) bulky hydrogels with micro/nano-porosity [20,21]; (3) bulky hydrogels with phase-separated structure [22,23]; (4) adjusting hydrophilic/hydrophobic balance of bulky hydrogels [24,25]. Besides, the most important factors to dominate the performance of responsive hydrogels are their chain structural parameters, such as the cross-linkage density and network architecture. Remarkably, a hydrogel with dangling chains grafted onto a cross-linked network, namely comb-type grafted hydrogel [26] has been developed. Compared with the normal hydrogels with only network component, the comb-type grafted hydrogels with network-graft architecture are endowed with improved molecular mobility due to dangling chains, and thus exhibit rapid conformational adjustment of grafting chains in response to environmental stimuli. Okano *et al.* obtained different comb-type grafted hydrogels based on poly(*N*-isopropylacrylamide) (PNIPAM) and symmetrically investigated their rapid deswelling behavior in response to temperature variation [26,27,28,29].

Generally, the comb-type grafted hydrogels with network-graft architecture have been prepared through three different routes. 1) The first is post-grafting polymerization from the network. Different groups utilized *γ*-radiation to generate polymeric radicals in the network and initiate grafting polymerization of vinyl monomers, resulting in the comb-type grafted hydrogels of the poly(acrylic acid) (PAA) network grafted with poly(4-vinylpiridine) [30], the PAA network grafted with PNIPAM [31,32] and the PAA network grafted with poly(acryloyl-l-prolinemethyl ester) [33]. However, those grafting polymerizations are badly controlled considering the grafting chain length. After the introduction of 2-bromoisobutyrate groups into the cross-linked poly[(2-(2-methoxyethoxy)ethyl methacrylate-*co*-oligo(ethylene oxide) methyl ether methacrylate-*co*-2-hydroxyethyl methacrylate-*co*-ethylene glycol dimethacrylate) [P(MEO2MA-OEOMA-HEMA)] hydrogels prepared from ATRP, the following “graft-from” ATRP of MEO2MA and OEOMA produced comb-type grafted hydrogels [34]. Thus, the thermo-responsive properties could be customized by changing the grafting density, grafted chain length, and the chain composition. 2) The second route is post-coupling reaction onto the network. Through the esterification and amidation of semi-telechelic side-chains with the network, we can derive the alginate network grafted with polyacrylamide [35], the alginate network grafted with PNIPAM [36], and the chitosan network grafted with PNIPAM [37,38]. The graft-onto method offers the opportunity to adjust the length and density of grafting chains. 3) The third route is graft-through copolymerization. Firstly, Okano *et al.* used macromonomers prepared by common radical polymerization to obtain comb-type grafted PNIPAM-based hydrogels [25,26,27,28,29,39,40]. Similarly, the comb-type grafted hydrogels of the poly(*N*,*N*-diethylacrylamide-*co*-2-dimethylaminoethyl methacrylate) [P(DEA-*co*-DMAEMA)] network grafted with P(DEA-*co*-DMAEMA)[41], the P(NIPAM-*co*-DMAEMA) network grafted with P(NIPAM-*co*-DMAEMA) [42], the P(DEA-*co*-AA) network grafted with PDEA [43] and the P(NIPAM-*co*-AA) network grafted with PNIPAM [44] have been obtained. However, common radical polymerization has bad controllability in the molecular weight of the obtained macromonomers. Through RAFT polymerization, Zheng *et al.* obtained the hydrogels of the PNIAPM network grafted with PAA by using trithiocarbonate-terminated PAA as a macromolecular chain-transfer agent.[22] In the above cases, the cross-linkage density can be controlled by the amount of small molecular cross-linker, such as *N*,*N*-methylenebisacrylamide. In our previous report, we developed a macro-cross-linker of PNIPAM with two azido groups at one chain and performed an azido-alkynyl click reaction with alkynyl-pending PDMAEMA to prepare network-graft hydrogels [45].

Network-graft hydrogels with different network and graft components exhibit dual responsive behavior. As for the P(NIPAM-*co*-AA) network grafted with PNIPAM, the swelling ratio in pure water rose with the amount of AA units in P(NIPAM-*co*-AA) and PNIAPM grafting chains while it decreased sharply at pH = 2.0 and temperature above 50 °C [44]. Liu *et al.* investigated the influence of grafting chain length on the responsive behaviors of comb-type grafted hydrogels with P(DEA-*co*-DMAEMA) as both the network and grafting chains, and found that longer grafting chains allowed a higher equilibrium swelling degree [46]. Some literatures also reported the influences of grafting chain fraction and grafting chain length on the responsive swelling behavior of other comb-type grafted hydrogels [27,34,47]. However, as noted by us, there is no report about a comparative study on the comb-type grafted hydrogels with reversed network-graft architecture.

In this manuscript, two series of comb-type grafted hydrogels from PDMAEMA and PNIPAM with reversed network-graft architectures were obtained through click reaction between alkynyl-pending copolymers of propargyl acrylate (ProA) and macro-cross-linkers with di-azido groups at one chain-end. These resultant hydrogels with reversed network-graft architecture exhibited different responsive behavior to temperature and pH as well as drug release behavior dependent on temperature and pH conditions.

## 2. Experimental

### 2.1. Materials

*N*-Isopropylacrylamide (NIPAM, 97%, Kohjin Co., Tokyo, Japan) was purified by recrystallization from a benzene/*n*-hexane mixture (65/35 *v*/*v*). *N*,*N*-Dimethylaminoethyl methacrylate (DMAEMA, Sigma-Aldrich, St. Louis, MO, USA) was passed through basic alumina column, then vacuum-distilled over CaH_2_ before use. 2-Bromoisobutyrol bromide (98%), 2-chloropropionyl chloride (98%) and bis(2-chloroethyl)amine hydrochloride (98%) were purchased from Alfa Aesar and used as received. Triethylamine was stirred with KOH for 12 h at room temperature, refluxed with benzene-4-sulfonyl-chloride, and distilled before use. *N*,*N*,*N'*,*N'*,*N’*-Pentamethyldiethylenetriamine (PMDETA, 98%, Sigma-Aldrich, St. Louis, MO, USA) was distilled over NaOH prior to use. Tris[2-(dimethylamino)ethyl]amine (Me_6_TREN) was prepared according to literature procedures [48]. The synthesis of dibenzyl-trithiocarbonate (DBTTC) and propargyl acrylate (ProA) can be referred to our previous report [45]. Tetrahydrofuran (THF) was dried over sodium/benzophenone and distillated just before use. *N*,*N*-Dimethylformamide (DMF) and *n-*hexane were dried and distilled over calcium hydride. Copper(I) bromide and copper(I) chloride was washed with glacial acetic acid, followed by washing with methanol and ethyl ether to remove impurities, then dried under vacuum and kept under N_2_ atmosphere. All other reagents were of analytical grade and used as received. Deionized water was used throughout the experiments. Aqueous solutions with pH = 4.0, 7.0 and 9.0 were prepared with NaOH or HCl solution.

### 2.2. Preparation of Macro-Cross-Linkers of PDMAEMA-(N_3_)_2_ and PNIPAM-(N_3_)_2_

Bis(2-chloroethyl)amine hydrochloride (6.60 g, 36.92 mmol), NaN_3_ (24 g, 369.2 mmol) and one pinch of KI were dissolved in deionized water (150 mL). The mixture in a 250-mL round-bottom flask was allowed to stir at 60 °C for 36 h. After that, the mixture was cooled to room temperature and NaOH aqueous solution (5 wt %) was added to adjust pH to 11.0. NaCl (10 g) was then added to the light yellow solution. The resulting mixture was extracted with benzene (5 × 100 mL). The organic phase was collected and dried over anhydrous MgSO_4_. After the filtration and rotary evaporation, the concentrated solution of bis(2-azidoethyl)amine hydrochloride was obtained and its content was measured by proton magnetic resonance (^1^H-NMR) spectroscopy (~25.8 wt %). (Cautions: excess concentration and vigorous shaking should be avoided.)

After the solution of bis(2-azidoethyl)amine hydrochloride (3.10 g, 20.0 mmol) and triethylamine (3.03 g, 30 mmol) in THF (90 mL) were cooled in an ice bath, a solution of 2-bromoisobutyryl bromide (6.90 g, 30.0 mmol) in THF (10 mL) was added dropwise. The addition lasted for more than 1 h, and the mixture was stirred at room temperature for 24 h. The resulting salt was filtered off and the filtrate was concentrated by rotary evaporation to obtain a yellow oily crude residue. The crude product was further purified by silica column chromatography eluting with CH_2_Cl_2_ to obtain colorless *N*,*N*-bis(2-azidoethyl)-2-bromoisobutyrylamide (AEBIA) (3.87 g, yield: 73%). (Caution: AEBIA is much stable but its solution is suggested used directly in the next step of ATRP).

PDMAEMA with two azido groups at one chain-end [PDMAEMA-(N_3_)_2_] was synthesized at the molar ratio of [monomer (3.14 g)]:[AEBIA (48.5 mg)]:[CuBr (20.1 mg)]:[PMDETA (24.2 mg)] = 70:1:1:1 at 25 °C in isopropyl alcohol (4.0 mL). The polymerization tube was equipped with a magnetic stirring bar and degassed by three freeze-pump-thaw cycles. After the tube was sealed under vacuum, the polymerization proceeded under stirring at 25 °C for 4 h. The tube was opened and the polymerization mixture was dialyzed against deionized water for 48 h using a dialysis bag with a cutoff molecular weight of 3500. PDMAEMA–(N_3_)_2_ was obtained by lyophilization (3.01 g, yield: 95%). The block length of the obtained product was determined to be 60 based on ^1^H-NMR analysis, and molecular weight distribution was obtained with gel permeation chromatograph (GPC). The samples of PDMAEMA*_n_*–(N_3_)_2_ with block length (*n*) of 40, 60 and 120 were obtained by adjusting the molar ratio of DMAEMA to AEBIA.

PNIPAM–(N_3_)_2_ with different block lengths was obtained through the similar route to the above, except that *N*,*N*-bis(2-azidoethyl)-2-chloropropionylamide (AECPA) was used as the initiator [45].

### 2.3. Preparation of Alkynyl-Pending Copolymers via RAFT Copolymerization

Linear alkynyl-pending copolymers were synthesized via RAFT copolymerization of NIPAM (or DMAEMA) and ProA using DBTTC as chain transfer agent. A typical synthetic procedure was followed. A solution of NIPAM (2.28 g, 20 mmol), ProA (0.11 g, 1 mmol), DBTTC (0.058 g, 0.2 mmol) and AIBN (0.003 g, 0.02 mmol) in anhydrous THF (5.0 mL) was added into a polymerization tube equipped with a magnetic stirring bar. The mixture was degassed by three freeze-pump-thaw cycles. After the tube was sealed under vacuum, the polymerization reaction proceeded under stirring at 80 °C for 24 h. The tube was opened and the reaction mixture was precipitated into cooled ether. P(NIPAM-*co*-ProA) (2.29 g, 95%) was obtained after the dryness under vacuum at 25 °C for 24 h.

Other copolymers of P(NIPAM*_p_*-*co*-ProA*_q_*) and P(DMAEMA*_p_*-*co*-ProA*_q_*) with different contents of ProA were prepared in the same way. The copolymer composition (*p* and *q*) was determined with ^1^H-NMR spectroscopy and the molecular weight distribution was characterized by GPC.

### 2.4. Synthesis of Network-Graft Hydrogels through Click Chemistry

Network-graft hydrogels with PNIPAM cross-linked network and PDMAEMA grafting chains (*n*-N/*g*-D) were synthesized through click reaction between P(NIPAM-*co*-ProA) and PDMAEMA–(N_3_)_2_ at near equivalence of azido and alkynyl groups. The cross-linking density was modulated with *p*/*q* in P(NIPAM-*co*-ProA). Thus, P(NIPAM_100_-*co*-ProA_10_) (139 mg), PDMAEMA_60_–(N_3_)_2_ (582 mg), CuBr (203 mg) and 2,2-bipyridine (57.8 mg) were dissolved in THF (3.0 mL). The mixture was deoxygenated by three freeze-pump-thaw cycles and sealed under vacuum. After the reaction at 50 °C without stirring for 24 h, the resulted hydrogel was purified by dialysis against THF/water (1/1) for 2 days and water for 3 days. The final dry hydrogel (0.53 g, 85%) was obtained by the lyophilization in a freeze drier (SIMENS FD5-3) for 48 h.

Network-graft hydrogels with PDMAEMA cross-linked network and PNIPAM grafting chains (*n*-D/*g*-N) were synthesized through click reaction between P(DMAEMA-*co*-ProA) and PNIPAM–(N_3_)_2_ through the above route [45].

### 2.5. Characterizations of Different Polymers

The apparent molecular weight and its polydispersity index were determined on a Waters 150C GPC instrument equipped with three Ultrastyragel columns (500, 10^3^ and 10^4^ Å) in series and a RI detector at 30 °C, using monodispersed polystyrene as calibration standard. DMF was used as the eluent at a flow rate of 1.0 mL/min. The ^1^H-NMR spectra were recorded at 25 °C on a Bruker AV300 NMR spectrometer (resonance frequency of 300 MHz) with CDCl_3_ as the solvent. Fourier transform infrared (FTIR) spectra were recorded on a MAGNA-IR 750 IR spectrometer in KBr pellets. UV–Vis measurement was performed on a Unico UV/vis 2802PCS spectrophotometer.

### 2.6. Swelling and De-Swelling of Network-Graft Hydrogels

For the study of swelling and de-swelling behavior of network-graft hydrogels at different pHs and temperatures, the dried hydrogel samples (diameter: 2 cm, thickness: 0.5 cm) were immersed into aqueous solutions with different pHs at 20 °C at the first. The swollen hydrogels were taken out from the solution at different time intervals. After wiping off the water on the sample surfaces with filter paper, the weights of the swollen hydrogels were recorded. The data presented are an average of three sampling. The swelling ratio (*R*_s_) was defined as followed:

(1) $$R_{s}=\frac{W_{s}-W_{d}}{W_{d}}\times 100\%$$

where *W*_s_ and *W*_d_ is the weight of swollen and dried hydrogel, respectively.

After the hydrogel sample reached the swelling equilibrium under certain condition (such as pH = 7.0, 20 °C), it was quickly transferred into another aqueous solution with different temperature or pH value (such as pH = 7.0, 40 °C). The de-swelling behavior was followed with the similar method as above. After the swelling or de-swelling process got to its own equilibrium state, the swelling/de-swelling cycles of the hydrogel between 20 and 40 °C at pH = 7.0 was obtained. The swelling/de-swelling cycles of the hydrogel between other conditions were carried out in the same way.

### 2.7. Drug Loading and Release of Ceftriaxone Sodium by Network-Graft Hydrogels

Ceftriaxone sodium, a third-generation and broad-spectrum cephalosporin antibiotic with high stability in the presence of β-lactamases, can exhibit potent antibacterial activity against both Gram-negative and Gram-positive bacteria. Furthermore, ceftriaxone sodium has good solubility in water and strong UV–Vis absorption at 292 nm. Thus, it was chosen as a model drug to examine the controlled release behavior of the dual sensitive hydrogels. The dried hydrogels (50.0 mg) were swollen in aqueous solution of ceftriaxone sodium (10.0 mg/g, 10.0 g) at 20 °C for 12 h. After the excess water on the surfaces was wiped off, the loading content of ceftriaxone sodium was estimated based on the weight difference of unloaded and drug-loaded dry gel.

After the drug loading, the drug release was carried out under different conditions. For example, after the drug-loaded hydrogel was immersed into the aqueous medium with pH of 4.0 at 40 °C, 2.0 mL solution was taken out for UV–Vis measurement at different intervals. The concentration of ceftriaxone sodium was determined at *λ* = 292 nm using the calibration built on the absorbance of ceftriaxone sodium solutions with known concentrations. At each sampling, 2.0 mL of the original medium was added to keep the constant volume of the medium. The data presented are an average of three measurements.

## 3. Results and Discussion

**Scheme 1:**
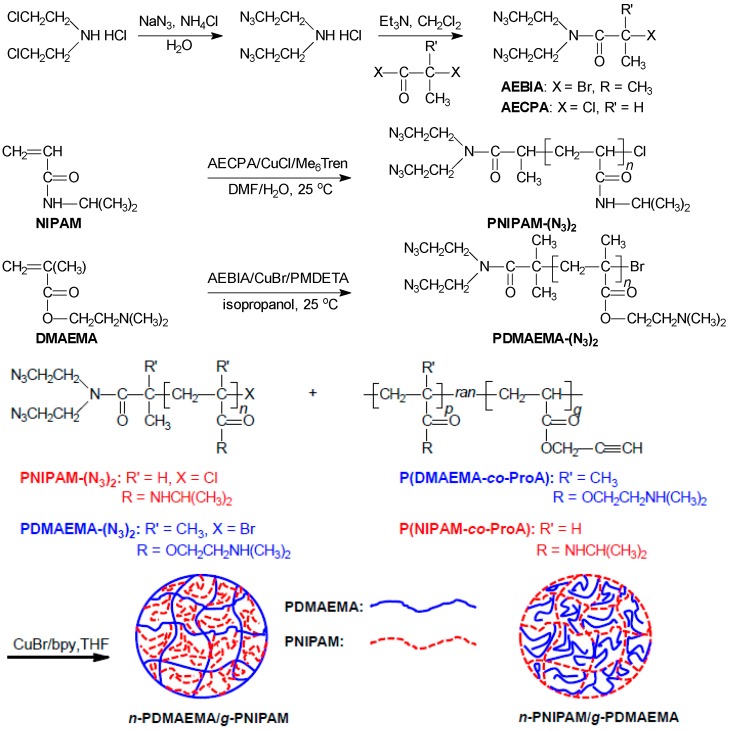
Schematic preparation of thermo- and pH-sensitive network-graft hydrogels of PNIPAM and PDMAEMA.

The network-graft hydrogels of PNIPAM and PDMAEMA with reversed network-graft architectures were synthesized as shown in Scheme 1. Firstly, macro-cross-linkers with azido groups at one chain-end were prepared by ATRP. Alkynyl-pending copolymers, as network precursors, were obtained by RAFT polymerization. The azide-alkyne click reaction between those two components resulted in the network-graft hydrogels with controlled structures.

### 3.1. Synthesis of Macro-Cross-Linkers with Di-Azido Groups at One Chain-End

Macro-cross-linkers with di-azido groups at one chain-end [PNIPAM–(N_3_)_2_ and PDMAEMA–(N_3_)_2_] were prepared through ATRP with different initiators and ligands in different reaction media to ensure the narrow molecular weight distribution of polymers. The integration of the AECPA/CuCl/Me_6_Tren initiation system and the DMF/H_2_O mixed solvent was chosen for the ATRP of NIPAM while AEBIA/CuBr/PMDETA and isopropanol were appropriated for the ATRP of DMAEMA.

**Figure 1:**
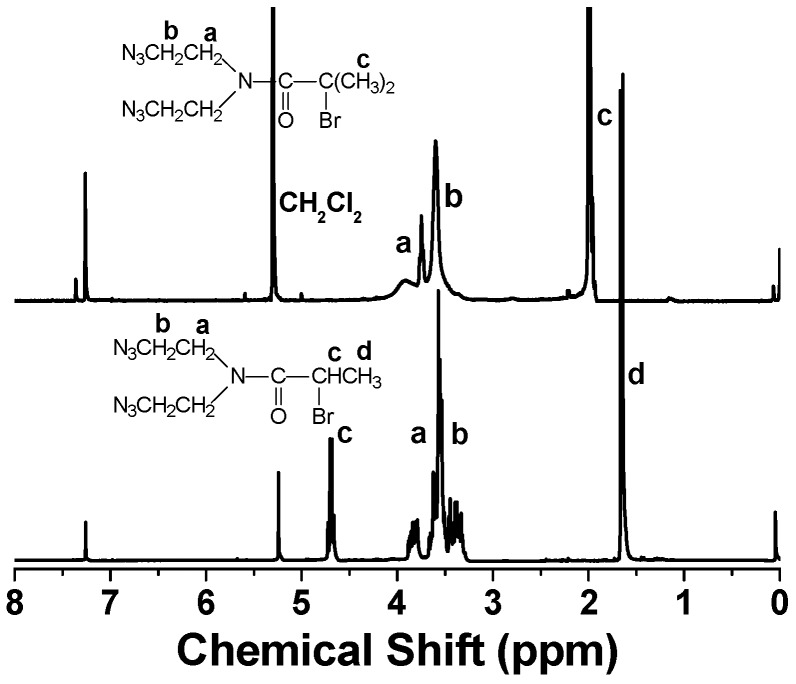
The ^1^H-NMR spectra of AECPA (lower) and AEBIA (upper) in CDCl_3_.

Figure 1 shows ^1^H-NMR spectra of AEBIA and AECPA. The methylene protons of C**H**_2_N(CO)– and C**H**_2_N_3_ have their signals in the range of 3.25~3.90 ppm and 3.25~4.10 ppm for AECPA and AEBIA, respectively. As for AEBIA, the signal of methyl protons is located at 2.00 ppm. AECPA has a signal of methyl protons at 1.64 ppm and a signal of methine protons at 4.73 ppm. The integration ratio of those characteristic signals is consistent with the chemical formulae. The signal at δ *=* 5.25 ppm belongs to the residual solvent of CH_2_Cl_2_, coming from the column chromatograph. FT-IR spectra of two ATRP initiators clearly reveal an absorbance peak of the azido group at ~2110 cm^−1^ (Figure S1).

**Figure 2:**
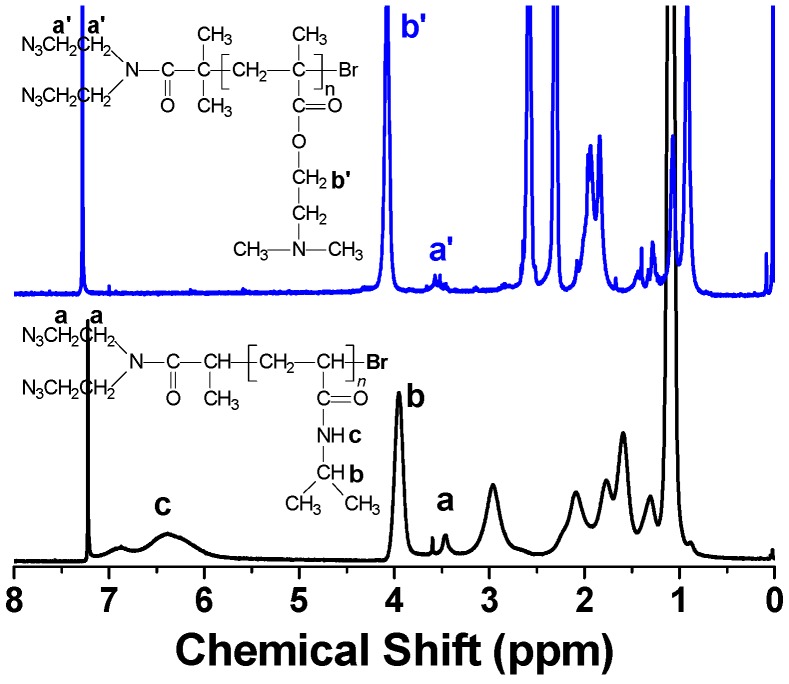
The ^1^H-NMR spectra of PDMAEMA_60_–(N_3_)_2_ (upper) and PNIPAM_60_–(N_3_)_2_ (lower) in CDCl_3_.

Figure 2 shows the typical ^1^H-NMR spectra of PDMAEMA–(N_3_)_2_ and PNIPAM–(N_3_)_2_, along with the assignment of characteristic signals. As for PDMAEMA–(N_3_)_2_, the proton signal of COOC**H**_2_CH_2_ appears at 4.01 ppm (b’), and the broad signal at 3.56 ppm (a’) is attributed to the methylene protons from the ATRP initiator of AEBIA. Based on the integration ratio of those two signals, the polymerization degree of PDMAEMA was determined to be 40, 60 and 120 for different PDMAEMA–(N_3_)_2_ macro-cross-linkers, respectively. PNIPAM–(N_3_)_2_ has its methine signal (b) from the isopropyl group at 3.85 ppm and the methylene signal (a) from AECPA at 3.46 ppm. The polymerization degree was calculated to be 60 and 100 for the two PNIPAM–(N_3_)_2_ macro-cross-linkers. GPC analysis of PDMAEMA–(N_3_)_2_ and PNIPAM–(N_3_)_2_ in DMF reveals the narrow molecular weight distribution (Figure S2). The details of structural characterization are summarized in Table 1.

**Table 1:** Characterization results of PDMAEMA–(N_3_)_2_ and PNIPAM–(N_3_)_2_ macro-cross-linkers.

Polymer	*M*_n,NMR_	*M*_n,GPC_	*M*_n_/*M*_w_ by GPC
PDMAEMA_40_–(N_3_)_2_	6,400	6,850	1.13
PDMAEMA_60_–(N_3_)_2_	9,600	10,100	1.15
PDMAEMA_120_–(N_3_)_2_	19,000	21,350	1.20
PNIPAM_60_–(N_3_)_2_	7,100	7,300	1.12
PNIPAM_100_–(N_3_)_2_	12,400	14,500	1.18

### 3.2. Preparation of Alkynyl-Pending Copolymers of P(NIPAM-co-ProA) and P(DMAEMA-co-ProA)

P(NIPAM-*co*-ProA) and P(DMAEMA-*co*-ProA) were synthesized through RAFT copolymerization, which could offer the narrow molecular weight distribution and uniform sequence distribution of the copolymers due to the dynamic equilibrium between the dormant and active species [49].

**Figure 3:**
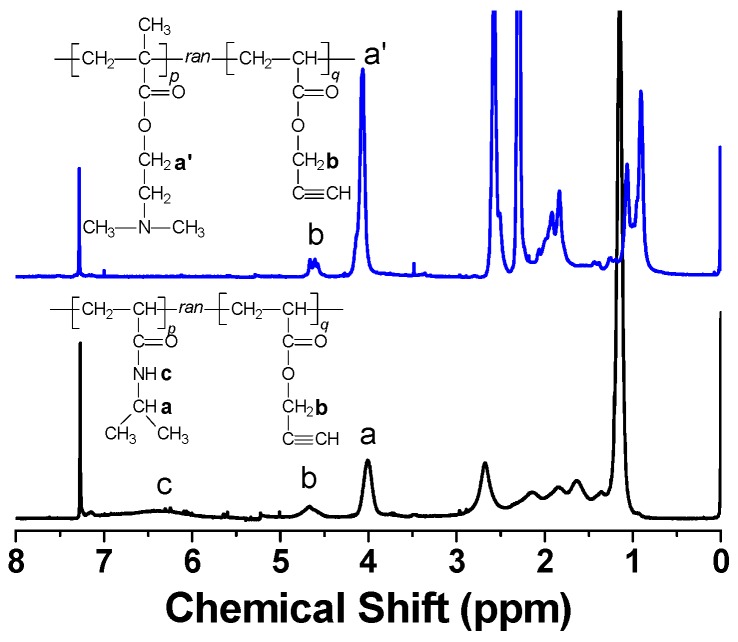
The ^1^H-NMR spectra of P(DMAEMA-*co*-ProA) (**upper**) and P(NIPAM-*co*-ProA) (**lower**) in CDCl_3_.

Their typical ^1^H-NMR spectra are demonstrated in Figure 3. The methylene protons of ProA units have the signal at about 4.65 ppm (b) for both kinds of copolymers. The characteristic signal of methine protons from the isopropyl group of P(NIPAM-*co*-ProA) and methylene protons next to the oxy-carbonyl group of P(DMAEMA-*co*-ProA) appears at 4.01 (a) and 4.06 ppm (a’), respectively. Based on the integration ratio of characteristic signals mentioned above, the copolymer composition was determined as *p/q* for P(DMAEMA*_p_*-*co*-ProA*_q_*) and P(NIPAM*_p_*-*co*-ProA*_q_*), where *p* was defined as 100. As summarized in Table 2, six ProA-containing copolymers have the molar ratio of NIPAM (or DMAEMA) to ProA being similar to that of the related monomer mixture. GPC traces of P(DMAEMA-*co*-ProA) and P(NIPAM-*co*-ProA) reveal the symmetric distribution of molecular weight for all the polymers (Figure S2) and the data are listed in Table 2.

**Table 2:** Characterization results of P(NIPAM-*co*-ProA) and P(DMAEMA-*co*-ProA).

Polymer	*p/q* (mole)	*M*_n,NMR_	*M*_n,GPC_	*M*_n_/*M*_w_ (GPC)
P(NIPAM_100_-*co*-ProA_5_)	100:5	12,100	13,400	1.24
P(NIPAM_100_-*co*-ProA_10_)	100:10	12,600	15,200	1.23
PNIPAM_100_-*co*-ProA_15_)	100:15	13,100	16,800	1.29
P(DMAEMA_100_-*co*-ProA_5_)	100:5	32,100	35,700	1.44
P(DMAEMA_100_-*co*-ProA_10_)	100:10	33,800	36,200	1.52
P(DMAEMA_100_-*co*-ProA_15_)	100:15	35,200	34,700	1.42

### 3.3. Synthesis of pH- and Thermo-Responsive Network-Graft Hydrogels with Reversed Architectures

The dual thermo- and pH-sensitive network-graft hydrogels composed of PNIPAM and PDMAEMA were formed through click chemistry between alkynyl-pending copolymers and diazido-capped polymers. Alkynyl-pending copolymers, such as P(DMAEMA-*co*-ProA) and P(NIPAM-*co*-ProA), built up the network component and their alkynyl content could modulate the cross-linkage density. Diazido-capped polymers, such as PNIPAM–(N_3_)_2_ and PDMAEMA–(N_3_)_2_, were used as the macro-cross-linker and grafting chains. High efficiency of the copper-catalyzed azide-alkyne click reaction would ensure the formation of network-graft architectures. To further guarantee almost all macro-cross-linkers participated in the reaction, alkynyl-pending copolymers had a slightly larger amount of alkynyl groups than azido groups in the diazido-capped polymers.

**Figure 4:**
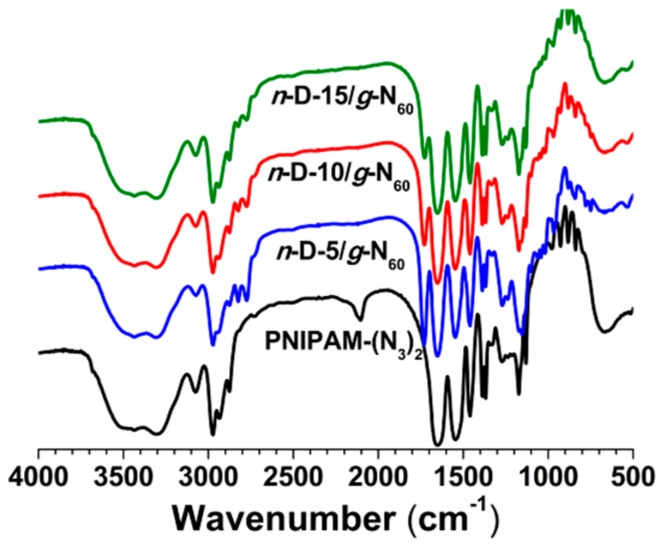
FT-IR spectra of network-graft hydrogels from P(DMAEMA-*co*-ProA) and PNIPAM_60_–(N_3_)_2_.

P(DMAEMA_100_-*co*-ProA_15_) and PNIPAM_60_–(N_3_)_2_ produced the hydrogels with the PDMAEMA network and PNIPAM grafting chains, named *n*-D-15/*g*-N_60_, while P(NIPAM_100_-*co*-ProA_10_) and PDMAEMA_40_–(N_3_)_2_ produced the hydrogels with reversed network-graft architecture, concurrently named *n*-D-10/*g*-N_40_. With the longest macro-cross-linkers of PDMAEMA_120_–(N_3_)_2_ and PNIPAM_100_–(N_3_)_2_, the formation of hydrogel was not observed, which should be the result from the spatial hindrance of grafting chains to retard the click reaction of azido end-groups. After purification by washing with water thoroughly, the hydrogels have no obvious band of azido characteristic absorbance at 2100 cm^−1^ in FTIR spectra as demonstrated in Figure 4.

Figure 4 shows the FT-IR spectra of *n*-D-5/*g*-N_60_, *n*-D-10/*g*-N_60_ and *n*-D-15/*g*-N_60_ from P(DMAEMA-*co*-ProA) with different ProA content compared to that of PNIPAM_60_–(N_3_)_2_. Besides the absence of azido characteristic absorbance, there exist two carbonyl vibration bands, respectively, for the ester bonds of ProA and DMAEMA residues at 1730 cm^−1^ and the amide bond of NIPAM units at 1651 cm^−1^. The gradual decrease in the magnitude ratio of A_1730_/A_1651_ from *n*-D-5/*g*-N_60_ to *n*-D-15/*g*-N_60_ suggests the increase in the PNIPAM content of three network-graft hydrogels, being consistent with the charged amount of PNIPAM_60_–(N_3_)_2_. As for the hydrogels of *n*-N-5/*g*-D_60_, *n*-N-10/*g*-D_60_ and *n*-N-15/*g*-D_60_ from P(NIPAM-*co*-ProA) and PDMAEMA_60_–(N_3_)_2_, the relative magnitude of the amide carbonyl bond compared with the ester carbonyl bond decreased with the amount of PDMAEMA_60_–(N_3_)_2_ charged initially (Figure S3).

### 3.4. Swelling/De-Swelling Behaviors of Network-Graft Hydrogels Dependent on Temperature Change

The present network-graft hydrogels are composed of PNIPAM and PDMEMA, offering them both thermal and pH responses. Therefore, those hydrogels have the ability to swell and de-swell in different conditions of temperature and pH value. The previous reports have revealed that those network-graft hydrogels behave responsively due to the cooperative variation of the network main-chain and grafting chains [22,27,44,45,46,47]. However, the network-graft architecture was not concerned with their response in the previous literatures. It should be expected that changing the architecture of comb-type grafted hydrogels in reverse could lead to a difference in the swelling/de-swelling behavior. Thus, *n*-N/*g*-D_60_ hydrogels are compared with our previous *n*-D/*g*-N_60_ hydrogels to investigate the influence of the network-graft architecture.

**Figure 5:**
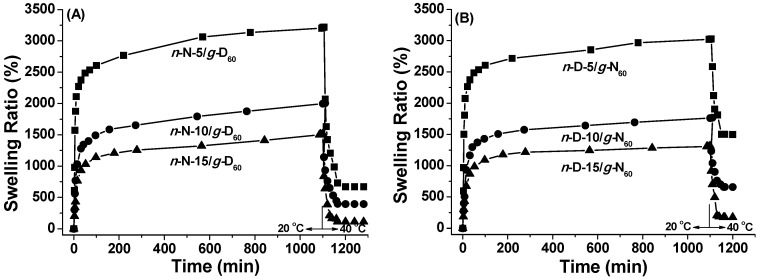
Swelling-deswelling kinetics of *n*-N/*g*-D_60_ (**A**) and *n*-D/*g*-N_60_ (**B**) hydrogels in deionized water at 20 and 40 °C (pH = 7.0).

Figure 5A shows the swelling and de-swelling kinetics of network-graft hydrogels of *n*-N/*g*-D_60_ hydrogels in neutral water (pH = 7.0) from 20 to 40 °C. All three hydrogels have excellent ability to uptake water at 20 °C and pH = 7.0, with saturated swelling ratios of 1900%, 2600% and 3700%, respectively. The values of the swelling ratios are a little higher than those of our previous *n*-D/*g*-N_60_ hydrogels with the same cross-linkage density and grafting chain-length (Figure 5B). Within about 10 min, all the hydrogels reach a highly swollen state, with an 80% saturated swelling degree. With the increase of cross-linkage density, the saturated swelling ratio of those hydrogels decreases obviously in spite of the increase or decrease of PNIPAM content. Keeping in mind that with the increase of cross-linkage density PNIPAM content decreases in *n*-N/*g*-D_60_ hydrogels while it increases in *n*-D/*g*-N_60_ hydrogels, PNIPAM is more hydrophilic than PDMAEMA at this condition of pH and temperature.

After the swelling equilibrium at 20 °C, the hydrogels were kept at 40 °C. It is observed that the de-swelling equilibrium for both the hydrogels with reversed network-graft architectures could be achieved in 60 min. It should be assigned to the response of PNIPAM segments. At 40 °C, the temperature above lower critical solution temperature (LCST) of PNIPAM, the PNIPAM segments collapse and the absorbed water is excluded from the hydrogels. The percent of excluded water increases from 79%, 80% to 93% for *n*-N/*g*-D_60_ hydrogels with the increase of cross-linking density, although grafting PDMAEMA chains still remain hydrophilic and their weight percent keeps increasing. As for the previous *n*-D/*g*-N_60_ hydrogels, the excluded water under the same condition occupies the weight percent in all the absorbed water, such as 50%, 63% and 86%, which also increase with the cross-linking density but are lower than *n*-N/*g*-D_60_ with reversed network-graft architecture. At the same cross-linkage density, *n*-D/*g*-N_60_ hydrogels have higher PNIPAM content than *n*-N/*g*-D_60_ hydrogels. The phenomena indicate that the cross-linked network is the most important factor to determine the water-holding capacity of network-graft hydrogels.

Meanwhile, it can be distinguished that *n*-D/*g*-N_60_ hydrogels with PNIPAM grafting chains have a slightly shorter duration for complete de-swelling compared to *n*-N/*g*-D_60_ hydrogels with the PNIPAM network, suggesting that grafting chains might prompt the shrinkage rate of network-graft hydrogels.

### 3.5. pH-Dependent on Swelling/De-Swelling Behaviors of Network-Graft Hydrogels

**Figure 6:**
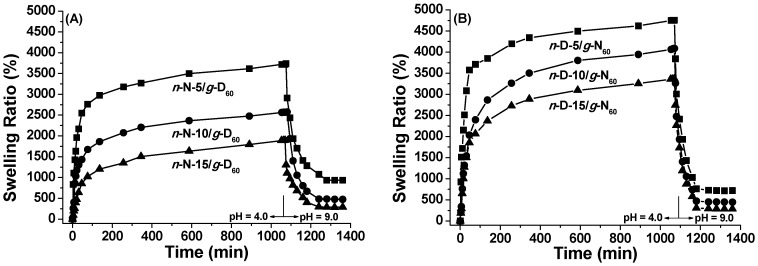
Swelling-deswelling kinetics of *n*-N/*g*-D_60_ (**A**) and *n*-D/*g*-N_60_ (**B**) hydrogels in deionized water at pH = 4.0 and 9.0 (20 °C).

The network-graft *n*-N/*g*-D_60_ hydrogels with different cross-linkage densities show similar pH-dependent swelling/de-swelling behavior at 20 °C in the aqueous solutions with two pH values, as seen in Figure 6A. The dependence of the swelling ratio of *n*-N/*g*-D_60_ hydrogels on pH value should be attributed to the presence of tertiary amino groups in the PDMAEMA segments. As reported, the p*K*_a_ of PDMAEMA is 7.0–7.3 [50]. In acidic medium, the amino groups in the network-graft hydrogels are ionized, which generates the electrostatic repulsion and breaks hydrogen bonding among polymer segments. Therefore, a higher swelling ratio could be obtained for the hydrogels. On the contrary, in alkalic medium, PDMAEMA segments are less hydrophilic from the de-ionization and become associated with each other. At the same time, hydrogen bonding would exist intensely in the polymer segments of the network-graft hydrogels. As a result, the hydrogel network becomes compact and the swelling ratio is lowered.

It should be mentioned that the swelling ratio reaches the saturated values of 3720%, 2780% and 1890% at pH = 4.0 for *n*-N-5/*g*-D_60_, *n*-N-10/*g*-D_60_ and *n*-N-15/*g*-D hydrogels, respectively, all of which are higher than those at pH = 7.0. Although the content of PDMAEMA in the hydrogels is increased, the saturated swelling ratio keeps going down with the cross-linkage density, also suggesting that the network main chain should be the predominant parameter over water-capture ability for network-graft hydrogels. After the saturated hydrogels are transferred into the aqueous solution at pH = 9.0 and 20 °C, the shrunk hydrogels reach their de-swelling equilibrium while the swelling ratio falls down to 940%, 480% and 390%, respectively. However, the equilibrium swelling ratios of the three *n*-D/*g*-N_60_ hydrogels (Figure 6B) have higher values for the swelling stage and lower values for the de-swelling stage. This comparison suggests that the network-graft architecture with the PDMAEMA cross-linked network and PNIPAM grafting chains more likely favors pH-induced sensitivity over temperature-induced sensitivity.

**Figure 7:**
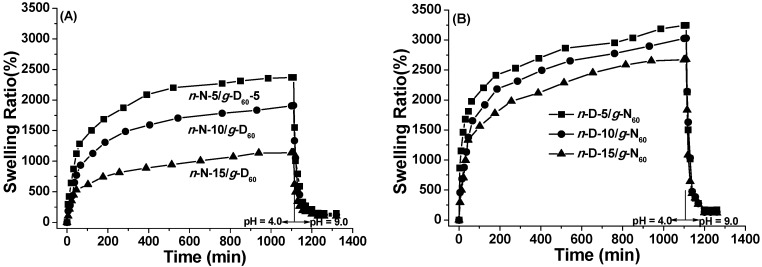
Swelling-deswelling kinetics of *n*-N/*g*-D_60_ (**A**) and *n*-D/*g*-N_60_ (**B**) hydrogels in deionized water at pH = 4.0 and 9.0 (40 °C).

When the dried hydrogels are kept sequentially in the aqueous solutions with pH of 4.0 and 9.0 at 40 °C, the swelling/de-swelling behavior is quite similar to that at 20 °C, as shown in Figure 7. However, the swelling ratios at 40 °C are rather lower than those at 20 °C, due to the chain collapse of PNIPAM segments. Moreover, the water-excluded amount increased much more sharply and reached a much lower value, indicating that high temperature and pH bring out the rapid and complete de-swelling of these dually responsive hydrogels with reversed network architecture.

To overview the results of the swelling and de-swelling behavior of network-graft hydrogels with different architectures, the equilibrium swelling ratios in swelling and de-swelling stages are listed in Table 3. Note that the macro-cross-linker with two azido groups at one chain-end also acts as the grafting chains in the comb-type grafted hydrogels, and thus the weight percent of the grafting chains increases with the cross-linking density. From the data in Table 3, the water-holding capacity of the network-graft hydrogels is mostly dependent on the cross-linking density and main-chain component more than other structural parameters. Both thermal- and pH-induced responsive behavior of the hydrogels has not only cross-linking density dependence but also relies on the network-graft architecture. The grafting chains are feasible for hydrogel shrinkage, leading to a higher de-swelling rate. The network nature does modulate the stimulus response, *i.e.*, the hydrogels with PDMAEMA as the network are more sensitive to pH variation while the hydrogels with PNIPAM as the network are more sensitive to temperature deviation. What can make the difference? The collapse of the network responding to the external stimulus directly leads to a reduced size of the gels, which corresponds to de-swelling behavior. This network process is direct and efficient, while grafted chain collapse causes de-swelling behavior in a complicated way. In principle, the single-chain collapse of grafted chains does not bring about great changes in the size of the hydrogel framework. More importantly, coordinated hydrophobic interaction among many grafted chains just forms efficient physical cross-link points, which leads to the noticeable collapse of the network and finally results in the hydrogel’s de-swelling process. It should be emphasized that both types of network-graft hydrogels have the highest equilibrium swelling degree at pH = 4.0 and 20 °C, and the lowest at pH = 9.0 and 40 °C, compared with other conditions of pH and temperature.

**Table 3:** Comparison of swelling behaviors of different comb-type grafted hydrogels made from PDMAEMA and PNIPAM.

Hydrogel	*R_s_* (%) at Equilibrium State					
	20 °C			40 °C		
	pH = 4.0	pH = 7.0	pH = 9.0	pH = 4.0	pH = 7.0	pH = 9.0
*n*-N-5/*g*-D_60_	3,720	3,220	940	2,370	670	150
*n*-N-10/*g*-D_60_	2,580	2,000	480	1,910	400	120
*n*-N-15/*g*-D_60_	1,890	1,520	290	1,140	110	100
*n*-D-5/*g*-N_60_	4,750	3,030	720	3,240	1,500	170
*n*-D-10/*g*-N_60_	4,090	1,770	460	3,030	660	130
*n*-D-15/*g*-N_60_	3,360	1,320	290	2,680	180	120

Furthermore, the swelling/de-swelling cyclization of the network-graft hydrogels was investigated, demonstrating that these hydrogels can be used circularly and have the dynamic responses of swelling-deswelling behavior via pH or temperature variation [45].

### 3.6. Loading and in Vitro Release of Ceftriaxone Sodium

The network-graft hydrogels with the well-controlled swelling behaviors can be applied as qualified carriers to trigger drug release. As the swelling/de-swelling properties can be tuned by temperature and pH, their performance in the controlled drug release system was investigated at various temperature and pH. Ceftriaxone sodium, one of the third-generation cephalosporin drugs, has been chosen as model molecule. Ceftriaxone sodium is dissolved well in water and has strong UV–Vis absorption at 292 nm, and thus its concentration in water can be easily determined.

**Figure 8:**
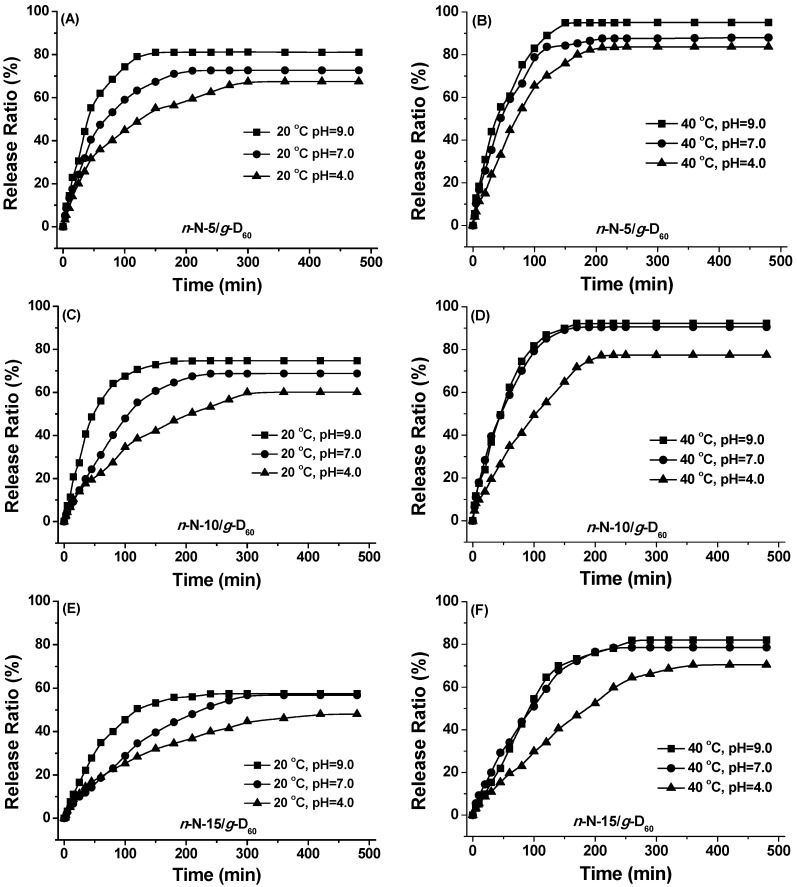
Drug release from *n*-N-5/*g*-D_60_ (**A** and **B**), *n*-N-10/*g*-D_60_ (**C** and **D**) and *n*-N-15/*g*-D_60_ (**E** and **F**) hydrogels under different temperatures and pHs.

The loading and drug release of ceftriaxone sodium with *n*-N/*g*-D_60_ have been investigated with the same procedure as in our previous report [45]. The amount of loaded drug was determined to be 16.8, 13.4 and 12.0 mg for the dry hydrogels (50 mg) of *n*-N-5/*g*-D_60_, *n*-N-10/*g*-D_60_ and *n*-N-15/*g*-D_60_, respectively. These values of drug-loaded amount decrease with the cross-linkage density, being the same as in our previous report. Additionally, the drug-loaded amount of the *n*-N/*g*-D_60_ hydrogel is a little higher than that of the *n*-D/*g*-N_60_ hydrogel with the same cross-linkage density. The release profiles of ceftriaxone sodium from the *n*-N/*g*-D_60_ hydrogels, as a function of time, are displayed in Figure 8.

Considering each figure for the release of ceftriaxone sodium from all three *n*-N/*g*-D_60_ hydrogels at each temperature, both the release rate and the maximum release amount increase with the increase of pH value. It should be caused by the shrinkage of the PDMAEMA segments of the hydrogels with pH as well as the static-electrical interaction between drug and PDMAEMA. Comparing Figure 8A,C,E with Figure 8B,D,F, ceftriaxone sodium releases at 40 °C much faster than at 20 °C, considering the time needed for the maximum release. At the same time, the maximum release amount also rises when the temperature goes up from 20 to 40 °C. The collapse of PNIPAM segments at the temperature above LCST should contribute to this observation. Cross-linking density also plays its role in the control of the drug release. The lower the cross-linking density is, the higher the release rate and the maximum release amount are. With a higher cross-linking density, the *n*-N/*g*-D_60_ hydrogel has a smaller pore size and lower shrinkage ability, leading to difficulty in drug emigration. These promising results suggest that the drug release can be easily mediated by temperature, pH value and hydrogel chemical structures.

## 4. Conclusions

Dual thermo- and pH-responsive comb-type grafted hydrogels were synthesized via the combination of ATRP polymerization with a diazido-capped initiator to obtain macro-cross-linkers [PDMAEMA–(N_3_)_2_ and PNIPAM–(N_3_)_2_], and via RAFT copolymerization of ProA with other vinyl monomers to produce alkynyl-pending copolymers [P(NIPAM-*co*-ProA) and P(PDMAEMA-*co*-ProA)] as network precursors as well as the azido-alkynyl click reaction between the diazido-capped macro-cross-linker and alkynyl-pending copolymers to form comb-type grafted hydrogels. Thus, the network-graft structure of the obtained hydrogels could be well controlled with regard to cross-linkage density, grafting density, grafting chain length and network-graft architecture. The final hydrogels exhibited temperature- and pH-induced response behavior with high saturated swelling ratio, rapid de-swelling and swelling-deswelling reproducibility under different conditions. We found that the swelling-deswelling ability can be efficiently tuned by varying the cross-linking density of the hydrogels. The comb-type grafted hydrogels with the architecture of the PNIPAM network and PDAMEMA grafting chains demonstrated stronger thermal response and weaker pH response compared with the comb-type grafted hydrogels with the reversed architecture of the PDMAEMA network main chain and PNIPAM grafting chains. The loading and releasing of ceftriaxone sodium in diverse circumstances confirmed the feasibility of the hydrogels as a stimulus-responsive drug delivery system. The release rate and amount of encapsulated drug molecules can be adjusted by the temperature and pH of the surrounding environment, along with the hydrogel structure.

## Supplementary Materials

Supplementary materials can be found at www.mdpi.com/2073-4360/8/2/38/s1.
