# Supplementary material for: Comb-Type Grafted Hydrogels of PNIPAM and PDMAEMA with Reversed Network-Graft Architectures from Controlled Radical Polymerizations
Source: Polymers (Basel). 2016 Feb 1;8(2):38. doi: 10.3390/polym8020038 (PMC6432512; doi:10.3390/polym8020038)
Supplement: Supplementary file 1 [file polymers-08-00038-s001.pdf]

Comb-Type Grafted Hydrogels of PNIPAM and PDMAEMA with Reversed Network-Graft Architectures from Controlled Radical Polymerizations

Sheng-Qi Chen, Jia-Min Li, Ting-Ting Pan, Peng-Yun Li and Wei-Dong He

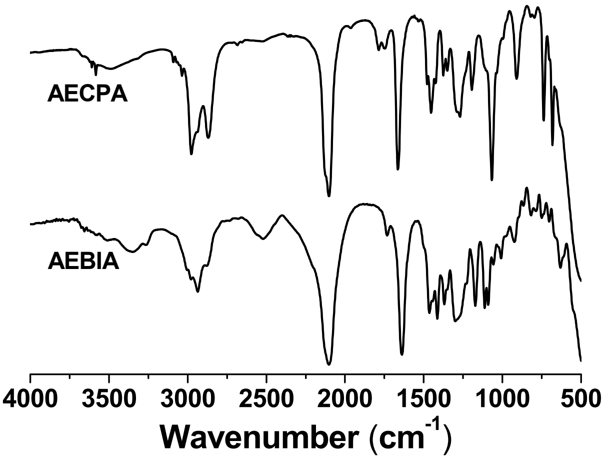

Figure S1. FT-IR spectra of AECPA and AEBIA.

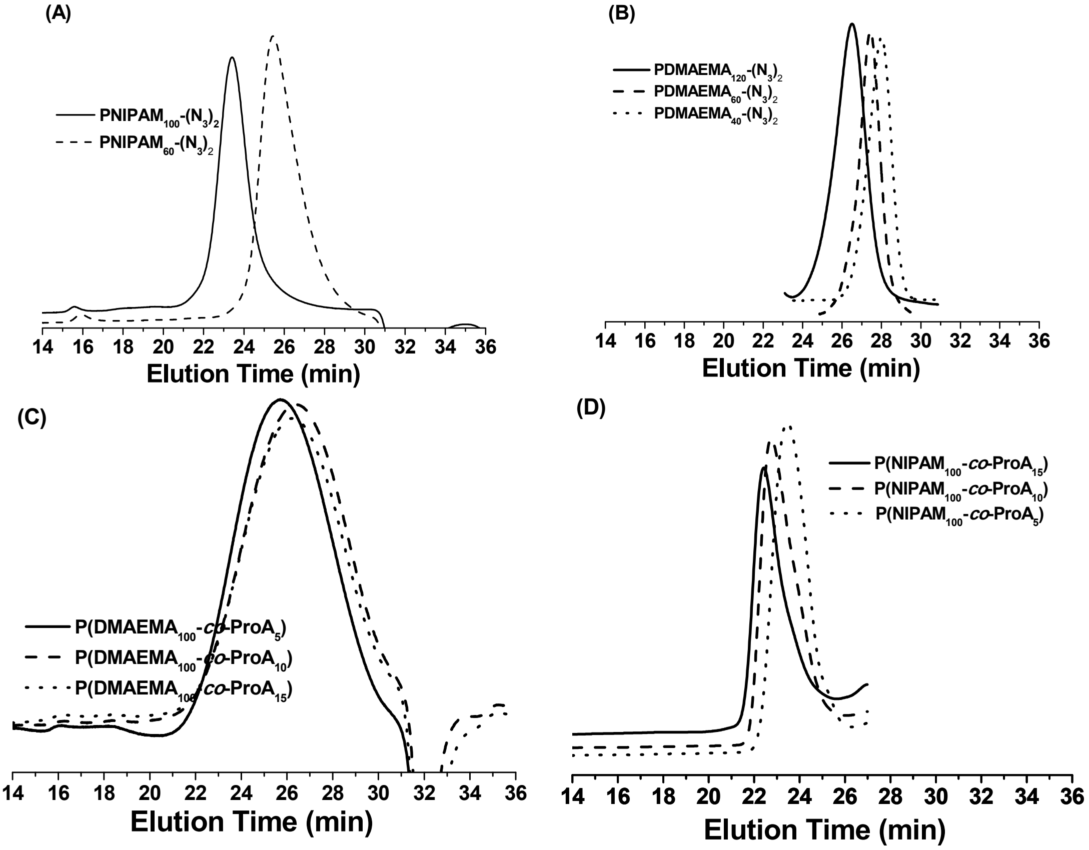

Figure S2. GPC traces of PNIPAM–$(N_3)_2$ (**A**), DMAEMA–$(N_3)_2$ (**B**), P(DMAEMA-*co*-ProA) (**C**) and P(PNIPAM-*co*-ProA) (**D**).

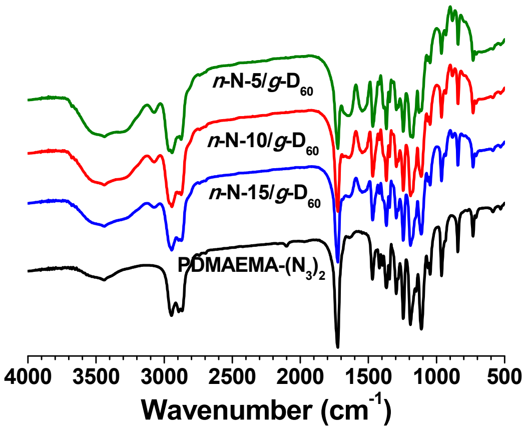

Figure S3. FT-IR spectra of network-graft hydrogels from P(NIPAM-*co*-ProA) and $PDMAEMA_{60}$–$(N_3)_2$.

© 2016 by the authors; licensee MDPI, Basel, Switzerland. This article is an open access article distributed under the terms and conditions of the Creative Commons by Attribution (CC-BY) license (http://creativecommons.org/licenses/by/4.0/).
